# Loss of KLF15 impairs endometrial receptivity by inhibiting EMT in endometriosis

**DOI:** 10.1530/JOE-23-0319

**Published:** 2024-04-17

**Authors:** Yaxiong Huang, Zihan Wang, Bin Li, Lina Ke, Yao Xiong, Yuanzhen Zhang

**Affiliations:** 1 Department of Reproductive Medicine center, Zhongnan Hospital of Wuhan University, Wuhan, Hubei Province, PR China; 2 Hubei Clinical Research Center for Prenatal Diagnosis and Birth Health, Wuhan Hubei Province, PR China; 3 Wuhan Clinical Research Center for Reproductive Science and Birth Health, Wuhan Hubei Province, PR China; 4 Department of Gynaecology and Obstetrics, Sinopharm Dongfeng Hospital, Hubei University of Medicine, Shiyan, Hubei Province, PR China

**Keywords:** endometrium receptivity, endometrial epithelial, KLF15, epithelial–mesenchymal transition, endometriosis

## Abstract

The impaired endometrial receptivity is a major factor contributing to infertility in patients with endometriosis (EM), but the underlying mechanism remains unclear. Our study aimed to investigate the role of Kruppel-like factor 15 (KLF15) in endometrial receptivity and its regulation in EM. We observed a significant decrease in KLF15 expression in the mid-secretory epithelial endometrial cells of EM patients compared to normal females without EM. To confirm the role of KLF15 in endometrial receptivity, we found a significantly reduced KLF15 expression and a significant decrease in embryo implantation number in the rat model via uterine horn infection with siRNA. This highlights the importance of KLF15 as a regulator receptivity. Furthermore, through ChIP-qPCR, we discovered that the progesterone receptor (PR) directly binds to KLF15 promoter regions, indicating that progesterone resistance may mediate the decrease in KLF15 expression in EM patients. Additionally, we found that the mid-secretory endometrium of EM patients exhibited impaired epithelial–mesenchymal transition (EMT). Knockdown of KLF15 upregulated E-cadherin and downregulated vimentin expression, leading to inhibited invasiveness and migration of Ishikawa cells. Overexpression KLF15 promotes EMT, invasiveness, and migration ability, and increases the attachment rate of JAR cells to Ishikawa cells. Through RNA-seq analysis, we identified TWIST2 as a downstream gene of KLF15. We confirmed that KLF15 directly binds to the promoter region of TWIST2 via ChIP-qPCR, promoting epithelial cell EMT during the establishment of endometrial receptivity. Our study reveals the involvement of KLF15 in the regulation of endometrial receptivity and its downstream effects on EMT. These findings provide valuable insights into potential therapeutic approaches for treating non-receptive endometrium in patients with EM.

## Background

Endometriosis (EM) is a benign gynecological disease that affects more than 10% of women of reproductive age (Practice Committee of the American Society for Reproductive Medicine 2012, Broi *et al.* 2019). Infertility is observed in approximately 30–50% of EM patients (Gavrizi & Skillern 2019, Sanchez *et al.* 2020). Despite the use of assisted reproductive technology therapy, patients with EM have a significantly lower clinical pregnancy rate compared to those without EM, and they also experience a higher rate of pregnancy-related complications (Breintoft *et al.* 2021, Corachan *et al.* 2021). Recent studies have indicated that the nonreceptive endometrium plays a crucial role in infertility among EM patients (Lessey & Kim 2017, Zachaki *et al.* 2021). Therefore, it is imperative to further investigate and understand the mechanisms underlying the reduced endometrial receptivity to effectively address infertility in EM. Glandular epithelial cells are pivotal in establishing endometrial receptivity, but the molecular mechanisms regulating the secretory phase transition of glandular epithelial cells need further investigation.

During the endometrial menstrual cycle, estrogen stimulates the proliferation of epithelial cells in the endometrium. In contrast, progesterone inhibits estrogen-induced epithelial cell proliferation, thus promoting the secretory transformation of endometrial epithelial cells (Ye 2020). Changes in the activity of estrogen and progesterone in the epithelial cells can contribute to various diseases, including endometriosis and endometrial polyps, all of which can affect embryo implantation (Kim *et al.* 2018, MacLean & Hayashi 2022). Kruppel-like factors (KLFs) are a family of transcription factors that contain a Cys2/His2 zinc finger domain. These factors bind to specific DNA motifs (CACCC or CGCCC) and are involved in embryogenesis, cell migration, and differentiation (Garcia-Nino & Zazueta 2021, Sun *et al.* 2022). Previous studies have shown that estrogen induces the expression of KLF4 and suppresses the expression of KLF15, leading to increased transcription of MCM2 and cell proliferation. However, when epithelial cells are treated with both estrogen and progesterone, KLF4 expression decreases, while KLF15 expression increases. The inverse expression pattern of KLF4 and KLF15 inhibits cell proliferation by suppressing the expression of MCM2 (Ray & Pollard 2012). Therefore, KLF15 may act as a downstream mediator of progesterone, which inhibits the proliferation of endometrial epithelial cells. In addition, Kim *et al.* (2015) have reported that KLF15 is positively regulated by ARID1A and PGR, which also inhibit epithelial cell proliferation during peri-implantation. Notably, the research did not further investigate the specific role of KLF15 in the remodeling of epithelial cell structure. The function of KLF15 in endometrial receptivity and embryo implantation is not fully understood. Further research is needed to elucidate the specific role of KLF15 in these processes.

In our current study, we examined the expression of endometrial epithelial KLF15 in patients with EM and compared it to normal females without EM. We observed a significant decrease in KLF15 expression in the mid-secretory endometrium of EM patients. In order to understand the mechanism underlying the regulation of KLF15, we performed ChIP-qPCR experiments to identify the target genes and upstream regulators of KLF15 in the endometrium. This investigation sheds light on the specific genes and factors that may influence KLF15 expression and its downstream effects. Our study provides valuable insights into the involvement of endometrial epithelium in infertility related to EM. By examining the expression of KLF15 and its potential regulatory network, we hope to contribute to a better understanding of the molecular mechanisms underlying impaired embryo implantation in EM patients.

## Materials and methods

### Ethics statement

The study was approved by the Ethics Committee of Sinopharm Dongfeng Hospital (LW-2022-021). Written informed consent was obtained from patients. All animal experiments were approved by the Institutional Animal Care and Use Committee and conducted following the 3R principle of experimental animals.

### Human endometrium samples

The data from GSE6364 were analyzed for kLF15 mRNA expression. It recruited 16 patients without endometriosis and 21 patients with endometriosis. We analyzed the KLF15 mRNA expression in their groups (Burney *et al.* 2007). We also recruited patients with or without endometriosis from Dongfeng General Hospital of Hubei Medicine University. Eutopic endometrial biopsy samples were collected from EM patients with infertility who had laparoscopic surgery (*n* = 12, six in the proliferative phase, six in mid-secretory phase). Normal endometrium was obtained from fertile patients free of EM (*n* = 12, six in the proliferative phase and six in the mid-secretory phase). All recruited patients were between the ages of 22 and 42 and had regular menstrual cycles. EM and the phase of endometrium were confirmed by pathological results. Furthermore, patients with submucous fibroids of the uterus, endometrial polyps, and hydrosalpinx endocrine, immune, and metabolic diseases were excluded. The control group consisted of patients who underwent laparoscopic hysterectomy for early-stage of cervical cancer. Tissue samples for immunohistochemistry analysis were immediately placed in 10% formalin and embedded in paraffin.

### Culture of Ishikawa cells and hormone treatment

The human endometrial adenocarcinoma cell line Ishikawa is commonly selected as a cell model to study the transformation of endometrial glandular epithelial cells from a non-receptive state to a receptive state (Buck *et al.* 2021). Ishikawa cells were purchased from the Cell Bank of the Chinese Academy of Science (Shanghai, China). Cells were cultured in RPMI-1640 media (Procell, Wuhan, China) containing 10% charcoal-stripped FBS (Clark Bioscience, Shanghai, China). After starvation treatment with 2% charcoal-stripped FBS for 24 h, medroxyprogesterone acetate (MPA) was added to the complete culture medium at final concentrations of MPA were 0 M, 1 × 10^−5^ M, 1 × 10^−7^ M, and 1 × 10^−9^ M. To study the role of progesterone receptor (PR) in regulating KLF15, 10 µM mifepristone was added to the culture medium for 24 h.

### Stable overexpression of KLF15

293T cells were transfected using the lentivirus packaging plasmid, pCDH-CMV-MCS-EF1-copGFP-T2A-Puro, along with the KLF15 overexpression plasmid. The transfection was performed using Lipofectamine™ 3000 reagent from Invitrogen, following the instructions provided by the manufacturer. After 72 h of transfection, the virus produced was concentrated using ultracentrifugation. The concentrated virus was then used to infect Ishikawa cells for a duration of 24 h.

### siRNA and overexpression plasmid transfection

To perform KLF15 knockdown in rats and *in vitro* cultured cells, siRNA targeting the rat or human KLF15 gene was obtained from Shanghai Gima Gene Company (Shanghai, China). For KLF15 knockdown in Ishikawa cells, the experimental group was transfected with 5 µL of KLF15 siRNA (100 nM), while the control group was transfected with a corresponding negative control siRNA (100 nM). For PGR overexpression, the fragment of the PGR gene was synthesized by Wuhan Jinkarui Biotechnology Co., Ltd. (Wuhan, China) and cloned into the pcDNA 3.1+ vector as a shuttling vector. Empty vectors were used as a negative control. Once the Ishikawa cells reached 80% confluence, either KLF15 siRNA or the pcDNA-PGR overexpression vector (1 µg) was transfected into the cells. The transfection process was carried out using Lipofectamine 2000, following the instructions provided by the manufacturer.

### Migration and invasion assay

For the migration assay, the cells were suspended in a concentration of 10^5^/mL without FBS. Then, 200 µL cell suspension without FBS were placed into a transwell chamber. Chambers were placed in 24-well plates by adding 500 µL of complete medium containing 20% FBS. The cells were then incubated for 24 h. After incubation, the chambers were stained with crystal violet for 10 min, and the non-cell-inoculated side was photographed under an inverted microscope.

For the invasion assay, Matrigel and medium were diluted at a ratio of 1:8, and 50 µL were added to the transwell chamber and dried in the incubator. For invasion assay, Matrigel and medium were diluted at a ratio of 1:8, and 50 µL Matrigel mixture were added to the transwell chamber and dried in the incubator. Two hundred microliters of the transfected cell suspension (10^5^/mL) without FBS were placed into a transwell chamber. Chambers were then placed in 24-well plates by adding 500 µL of complete medium containing 20% FBS. After incubating for 24 h, use a cotton swab to gently wipe away any remaining cells in the chamber. The chamber was then gently placed in 4% paraformaldehyde fixative and fixed for 15 min at room temperature. The chambers were stained with crystal violet for 10 min, then the chambers were stained with crystal violet for 30 min. According to the microscopic observation, the number of transmembrane cells in five fields was counted randomly under 200× magnification, and the average represents the invasiveness of cells in one chamber.

### Scratch wound healing assay

Wound-healing assays were conducted in six-well plates. The cells were plated in these plates and allowed to grow until they reached 100% confluency. After reaching full confluency, a straight scratch/wound was created in the cell monolayer using a sterile pipette tip. This scratch served as the starting point for cell migration. The cells were then incubated for 48 h to allow migration into the scratched area. During this time, the cells migrating from the leading edge of the scratch were photographed. Multiple views of each well were documented to capture the extent of cell migration. To ensure reliable results, four independent experiments were performed.

### Assessment of trophoblast spheroid attachment to Ishikawa monolayer

Ishikawa cells were cultured in six-well flat-bottom plates, forming a monolayer, and incubated at 37°C with 5% CO2. To model blastocysts, the embryonic trophoblast cell line JAR was used, as previously reported (Gou *et al.* 2019). JAR cells were subjected to constant temperature oscillation in an incubator, with a speed of 4 ***g***, at 37°C for 24 h. This process resulted in the formation of embryonic trophoblast spheroids. The spheroids were collected and suspended in a working solution that contained a fluorescent tracer for living cells, provided by YEASEN Biotech Co., Ltd (Shanghai, China). After incubating for 30 min, the working solution was removed through centrifugation, and fresh medium was added for further culture for 30 min. Subsequently, the culture media containing the JAR spheroids were filtered through a 70 μm cell mesh, allowing the collection of JAR spheroids larger than 70 μm in size. JAR spheres with fluorescence >70 μm were collected and resuspended to achieve uniform density. Following this, an equal volume of culture media (100 μL) containing the JAR spheroids was added to the Ishikawa cell monolayer, which had been stained with DAPI. Count the number of JAR spheroids in each well. After incubating for 1 h, the number of JAR spheroids attached to the Ishikawa cells was observed under a microscope. The attachment rate was calculated by dividing the number of attached spheroids by the total number of spheroids added to the Ishikawa cells and multiplying by 100%.

### Animals and endometriosis induction

Female Sprague-Dawley (SD) rats, 6-weeks-old, were obtained from Weitong Lihua Laboratory Animal Technology Co., Ltd. (Beijing, China). The rats were housed in a controlled environment with a 14 h light and 10 h darkness cycle, maintained at a temperature of 20−25°C and humidity level of 40−50%. Prior to modeling, all rats were adaptively fed for 1 week to acclimatize to the experimental conditions.

To induce the endometriosis rat model, a surgical procedure was performed. A 2 × 3 mm piece of the right distal end of the uterus horn, containing the intima, muscular layer, and serosal layer, was minced. One piece of uterine tissue was sewn onto the right abdominal wall, while the other piece was sewn near the right ovarian end. These implanted uterine tissues were fixed in the vicinity of the great vessels, with the intimal surface facing the abdominal cavity (Supplementary Fig. 1). The rats were then housed for a period of 3 weeks. To confirm the success of the model, hematoxylin and eosin (HE) staining was performed (Supplementary Fig. 2). A sham-operated group was included, where only the distal end of the right uterine horn was excised and sutured.

For fertility studies, adult female rats were paired with male rats. The morning of the observation of a vaginal plug was designated as day 1 of gestation (GD 1). Uterine samples from pregnant rats were collected on GD 1, GD 5, and GD 7. The number of litters after pregnancy was recorded for further analysis.

For KLF15 knockdown rat model, rats without endometriosis on GD4 were operated, 50 µL of KLF15 siRNA (10 µM) were injected into the left uterine horns, and 50 µL control siRNA (10 µM) were injected into the right uterine horns using a microinjector. The number of litters after siRNA injection was recorded on GD7 (Fig. 2). This allowed for assessing the potential effects of KLF15 knockdown on fertility and reproductive outcomes in the rat model.

### Immunohistochemistry

Endometrial tissues were fixed by 4% paraformaldehyde and embedded in paraffin. The sections were later deparaffinized and rehydrated in graded ethanol, and antigen retrieval was performed. For staining the sections, the UltraSensitive SP IHC Kit from Fuzhou Maixin Biotechnology Development Co., Ltd. (Minhou, China) was used following the instructions provided by the manufacturer. IHC staining was performed on paraffin sections with antibody against KLF15 (1:200; Abcam) and antibody against PGR (1:200, Fuzhou Maixin Biotechnology Development Co., Ltd.). To conduct semi-quantitative histologic scoring (*H*-score) analysis, ten fields were chosen from each immunohistochemical section. All of these slides were then subjected to analysis using Image J software.

### Immunofluorescence staining

Different groups of Ishikawa cells cultured in chamber slides were fixed in 4% paraformaldehyde, followed by permeabilization and blocking. Cells were then incubated with respective primary antibodies, including anti-vimentin antibody (1:2500, Proteintech, Hubei, China), anti-E-cadherin antibody (1:2000, Proteintech), overnight at 4°C. Cy3-labeled goat anti-rabbit IgG (1 μg/mL, Sigma) and goat anti-mouse IgG (1 μg/mL, Sigma) were used, respectively, to visualize the signal, and nuclei were stained with DAPI (1 μg/mL, Sigma). The cell pictures were observed under an inverted fluorescence microscopy imaging system (Olympus).

### Real-time RT-PCR analysis of mRNA expression

Total RNA was extracted using the EASY spin Tissue/Cell RNA Rapid Extraction Kit (Aidlab Biotechnologies Co., Ltd., Beijing, China) according to the manufacturer’s protocol. Total RNA (1 μg) from each sample was treated with DNase I and reverse transcribed using the Prime Script^TM^ RT reagent Kit (TaKaRa). The real-time PCR assay was performed using SYBR® Premix Ex-Taq™ (TaKaRa) with the Step One™ Real-Time PCR System (Life Technologies). The procedure for RT-qPCR was as follows: 95°C for 30 s to denature the DNA, followed by 95°C with a time of 10 s and 60°C for 30 s. In total, 40 cycles were performed. The primer sequences used are listed in Table 1. Experimental gene expression data were normalized to GAPDH gene. Expression levels of the samples were calculated using the 2^−ΔΔCt^ method. All measurements were performed at least in triplicate, and the average of the replication was used for statistical analysis.

**Table 1:** Primers used in RT-PCR.

Gene		Primer
**Human**		
Actin	F	5′-CGCTAACATCAAATGGGGTG-3′
	R	5′-TTGCTGACAATCTTGAGGGAG-3′
KLF15	F	5′-ATGCACAAATGTACTTTCCCT-3′
	R	5′-TCAGTTCACGGAGCGCACGGA-3′
PGR	F	5′-AATCATTGCCAGGTTTCGAA-3′
	R	5′- GCCCACTGACATGTTTGTAGGA-3′
E-cadherin	F	5′-CGAGAGCTACACGTTCACGG-3′
	R	5′-GGGTGTCGAGGGAAAAATAGG-3′
Vimentin	F	5′-AGTCCACTGAGTACCGGAGAC-3′
	R	5′-CATTTCACGCATCTGGCGTTC-3′
TWIST2	F	5′-CGCACCCAGTCGCTCAACG-3′
	R	5′-TCTTATTGTCCATCTCGTCGC-3′
**Rat**		
GAPDH	F	5′-CGCTAACATCAAATGGGGTG-3′
	R	5′-TTGCTGACAATCTTGAGGGAG-3′
Klf15	F	5′-GGAGGCTGAGGTCAAGGAGG-3′
	R	5′-GCACCTGCTTCCTGCTTCAC-3′
PGR	F	5′-TTACCATGTGGCAAATCCCAC-3′
	R	5′- TAGAAGCGTTGTGAACTGGGG-3′
E-cadherin	F	5′-TGAGGTCGGTGCCCGTATT-3′
	R	5′-CGTTGGTCTTGGGGTCTGT-3′
Vimentin	F	5′-AGGGGAGGAGAGCAGGATTT-3′
	R	5′-GTGTTCTTTTGGAGTGGGTG-3′

### Western blot

Total cell lysates were prepared in RIPA buffer supplemented with protease and phosphatase inhibitors, then resolved on 8–15% SDS-PAGE gels and electroblotted onto PVDF membrane. The membranes were blocked with a rapid blocking solution (Beyotime, Wuhan, China) and then incubated in primary antibodies prepared in a blocking buffer Beyotime). The membranes were blocked before incubating with anti-PGR (1:1000, Santa Cruz Biotechnology), anti-KLF15 (1:1000, Santa Cruz Biotechnology), anti-TWIST2 (1:1000, Abcam), anti-E-cadherin (1:1000, Santa Cruz Biotechnology), anti-vimentin (1:1500, Proteintech), and anti-GAPDH (1:2000, Proteintech) antibodies overnight at 4°C. On the second day, membranes were washed with PBST, followed by incubating with TBST for 2 h. Then they were incubated with horseradish peroxidase (HRP)-conjugated secondary antibodies for 1 h, and blots were developed with an Enhanced ECL chemiluminescence detection kit (Vazyme, Nanjing, China) and imaged using the Tanon 5200 ECL chemiluminescence imaging system. Densitometry was performed using ImageJ.

### Dual-luciferase reporter assay

Transfection of the firefly luciferase gene ligated to the KLF15 promoter was performed with pGL3 plasmids using lipofectamine transfection. A pRL-SV40 (Promega) control vector containing the Renilla luciferase gene was co-transfected for normalization of luciferase activity. Cells were transfected with these two vectors with or without MPA or PGR overexpression plasmid (pcDNA-PGR, synthesized by Wuhan Jinkarui Biotechnology Co., Ltd.). Twenty-four hours after transfection, cells were washed in PBS, and harvested using passive lysis buffer (Promega), and firefly and Renilla luciferase activities were measured using the GloMax 20/20 luminescence detector (Promega). The firefly luciferase activities were normalized to the activities of Renilla luciferase.

### Chromatin immunoprecipitation assay

ChIP-qPCR assays were performed using the Simple ChIP Plus Sonication Chromatin IP Kit (CST, #56383) as per the manufacturer’s protocol. Chromatin was fragmented by sonication. A 5-min 2-s open/1-s closed treatment period (sonication time was 5 min) and 30% amplitude were applied. For each ChIP reaction, 10 µg of chromatin were immunoprecipitated using 5 µL of antibodies against PGR (Santa Cruz Biotechnology) or KLF15 (Santa Cruz Biotechnology). The immunoprecipitated samples were then incubated at 4°C overnight with shaking. The sequences of the primers used for the PGR response element in the KLF15 gene and the KLF15 response element in the TWIST2 gene are provided in Table 2. Immunoprecipitation with normal rabbit IgG was performed as a negative control. The resulting signals were normalized to input DNA.

**Table 2:** Primers used in ChIP-qPCR.

	Primer
**Primers used for the PGR response element in *KLF15* gene**	
Primer1	AAAACGTCCCCTAGAACGGC
	TGAAGTTGAACCCGAGACCG
Primer2	TAAGAAATGGTCCCCGACACG
	GGACCGCCAGTGTAAGGAC
Primer3	GGCAAAACTGAAAGTGCCG
	AATAGTTGTCAGGAGCTAGGGC
**Primers used for KLF15 response element in *TWIST2* gene**	
Primer1	GCTGGATTATGCCTCTGTGAT
	TTTGGTATTTATTTGCTGGTAGTT
Primer2	CGCTGCACCACATCTGGAAG
	TAACCTCGCTCGGTGAGCCC
Primer3	GGCTCTCATTAACACCAGAGGCT
	GCTTCCAGATGTGGTGCAGCG

### Statistical analysis

Utilizing SPSS16, we analyzed data presented as mean ± s.d. One-way ANOVA facilitated comparison among multiple groups, while the *t-*test assessed differences between two groups, considering statistical significance at *P* < 0.05.

## Results

### KLF15 expression is decreased in EM patients with infertility

**Figure 1:**
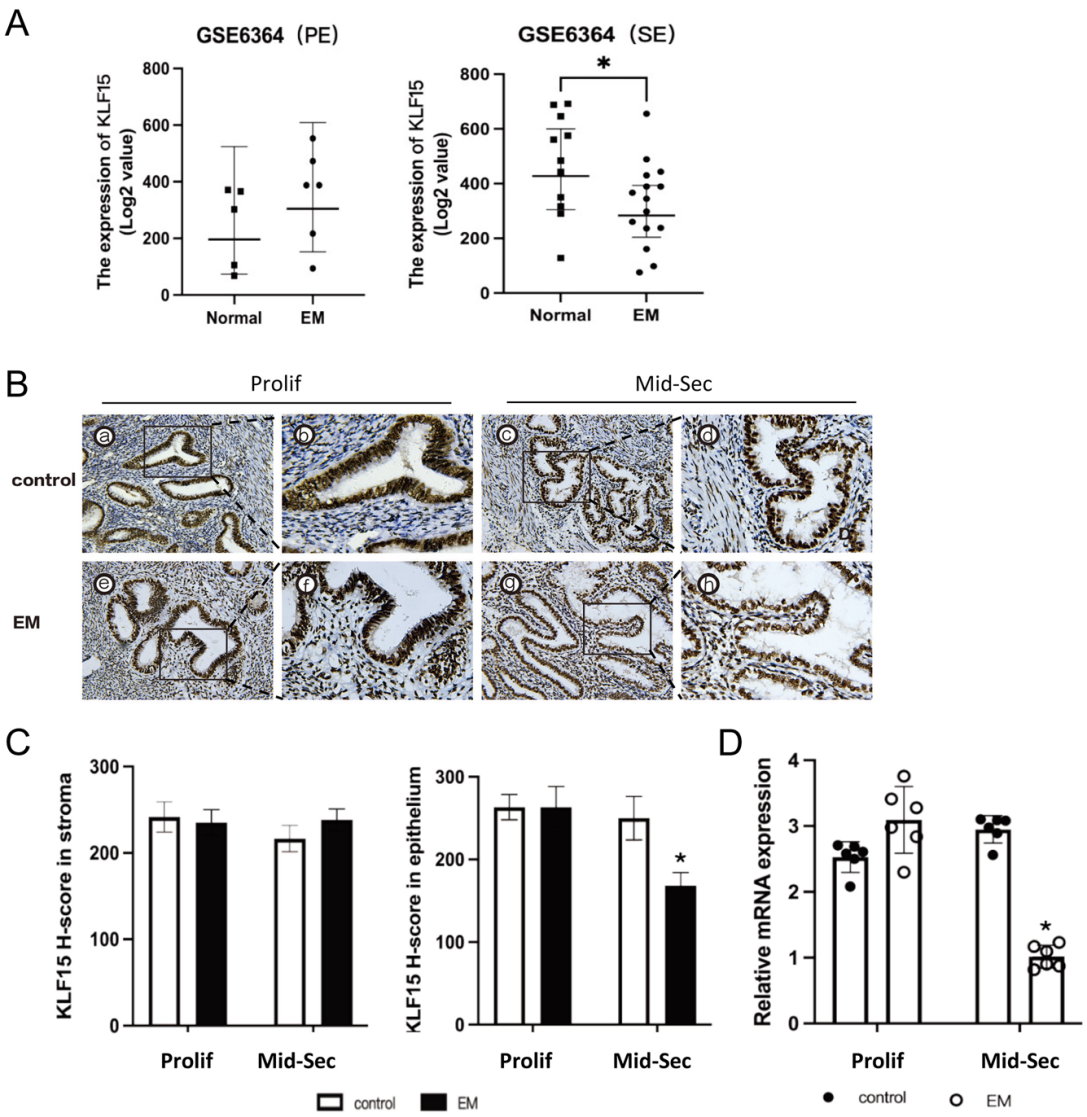
The expression pattern of KLF15 in endometriosis. (A) Analysis of KLF15 mRNA expression in proliferative and mid-secretory endometrium of healthy women of childbearing age with or without endometriosis using GSE6364 dataset. (B) Representative photomicrographs of KLF15 protein expression in EM and control group. The left photos were shown in 200× magnification, the right photos were shown in 400× magnification. (C) Immunohistochemical *H*-score of KLF15 proteins in proliferative (*n* = 6) and mid-secretory phase (*n* = 6) endometrium from women with (*n* = 12) or without (*n* = 12) endometriosis. (D) RT-qPCR analysis of KLF15 expression in different phases of endometrium in patients with (*n* = 12) or without endometriosis (*n* = 12). *P < 0.05, **P < 0.01, using one-way ANOVA. Prolif, proliferative phase; Mid-Sec, mid-secretory phase; EM, endometriosis. A full colour version of this figure is available at https://doi.org/10.1530/JOE-23-0319.

The results of the GSE: 6364 datasets (Burney *et al.* 2007) showed that KLF15 mRNA expression decreased in the eutopic mid-secretory endometrium of women with endometriosis (Fig. 1A). However, there were no significant differences in KLF15 mRNA expression in the proliferative phase. To further investigate this, we examined the protein expression of KLF15 in the endometrium of infertile women with endometriosis using immunohistochemistry. The immunohistochemistry results showed that KLF15 was primarily located in the nucleus and was abundant during both the proliferative and mid-secretory phases in the control group of fertile women (Fig. 1B). However, in the mid-secretory phase of epithelial cells in the eutopic endometrium from women with endometriosis, the *H*-score of KLF15 was significantly lower. There were no significant changes in the nuclear signal of KLF15 in stromal cells (Fig. 1C). In addition to the protein expression, the expression of KLF15 mRNA was also found to be decreased in women with endometriosis compared to the control group during the mid-secretory phase, as detected by RT-qPCR analysis (Fig. 1D).

### Reduced KLF15 expression causes embryo implantation failure

To investigate the expression pattern of KLF15 during embryo implantation in rats with EM, we generated an EM mouse model (Supplementary Figs. 1 and 2). We observed that KLF15 was upregulated on gestation day 5 (D5) and day 7 (D7) in both normal and sham operation control groups. However, in EM rats, KLF15 expression decreased after embryo implantation compared to control mice on D5 and D7 (Supplementary Fig. 3).

**Figure 2:**
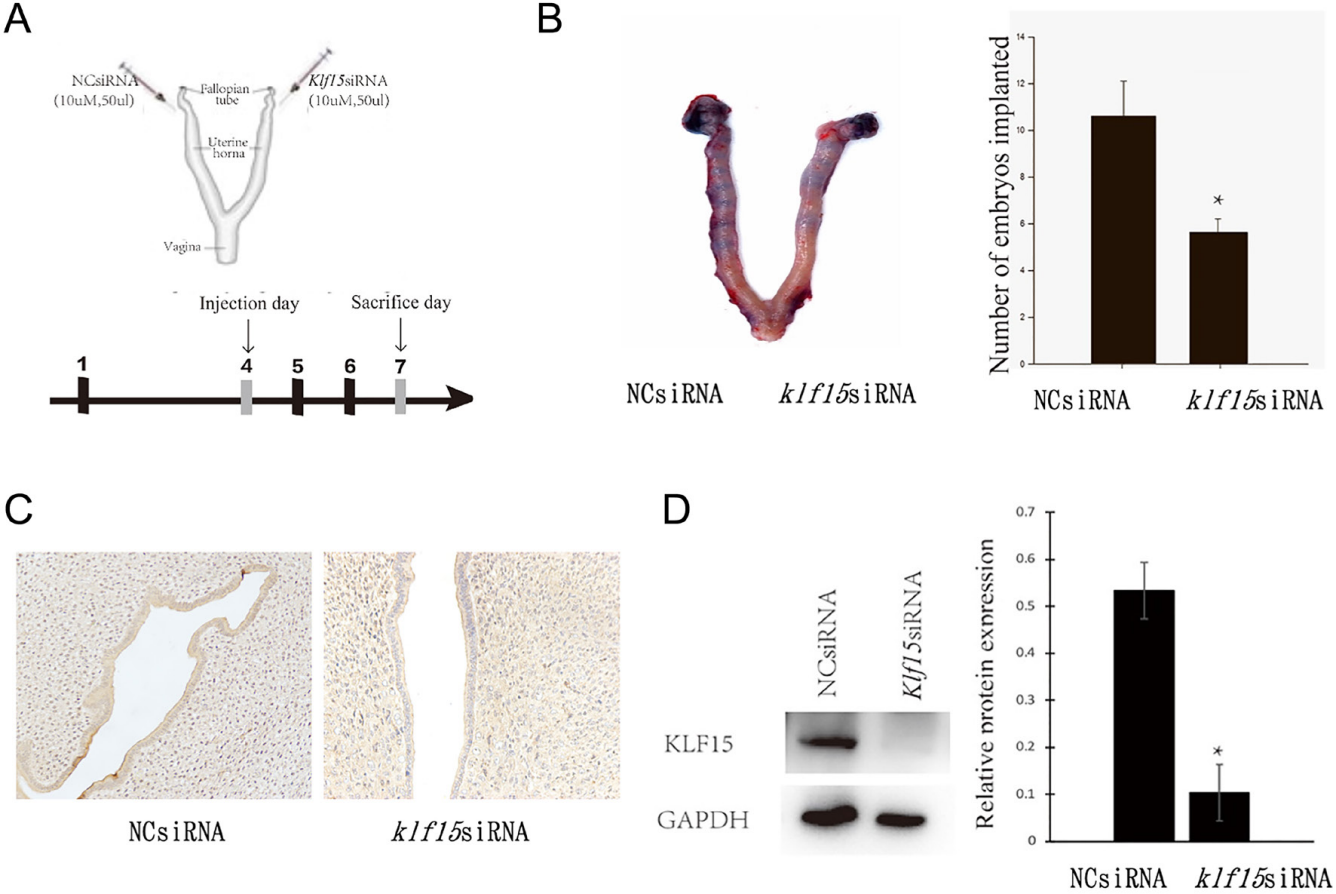
Attenuation of KlfF15 in rat models. (A) Establishment of the KLF15 knockdown rat model. On the 4th day of pregnancy, 50 µL 10 µM Klf15 siRNA were injected into the left uterine cavity from the uterine horn near the ovary, and the same volume of 10 µM NC-siRNA (negative control) was injected into the opposite side of the uterine horn as a control. The rats were sacrificed on the 7th day of pregnancy to observe the number of embryo implantation sites. (B) The decreased number of implantation sites after KLF15 siRNA injected into the left horn of uteri (*n* = 8). (C) Representative photomicrographs of KLF15 protein expression in rat uterus tissues injected with NC-siRNA or KLF15-siRNA. Photos are shown in 400× magnification. (D) The protein expression of KLF15 in rat uterus tissues injected with NC-siRNA or KLF15-siRNA (*n* = 8). *P < 0.05, using *t*-test. A full colour version of this figure is available at https://doi.org/10.1530/JOE-23-0319.

To evaluate the role of KLF15 in endometrial receptivity, we injected KLF15-siRNA into the left horn of the uterus on gestation day 4 (GD4), while the right horn received NC siRNA as a control (Fig. 2A). Western blot and IHC showed that the protein expression of KLF15 was reduced in rat uterus tissues injected with KLF15-siRNA (Fig. 2B). We found that the number of embryo implantations significantly reduced after KLF15 knockdown (Fig. 2C). Furthermore, we used an *in vitro* embryo implantation model to verify whether decreased KLF15 impaired the endometrial adhesive capacity of trophoblast cells. Ishikawa cells were stably transfected with KLF15-siRNA, and JAR spheroids (mimicking blastocysts) were cocultured with the Ishikawa cell monolayer. After 24 h, we counted the number of attached JAR spheroids, and it decreased in the KLF15-siRNA group (Supplementary Fig. 4). These results suggest that decreased KLF15 expression in Ishikawa cells might inhibit blastocyst attachment.

### Progesterone receptor regulates KLF15 expression

**Figure 3:**
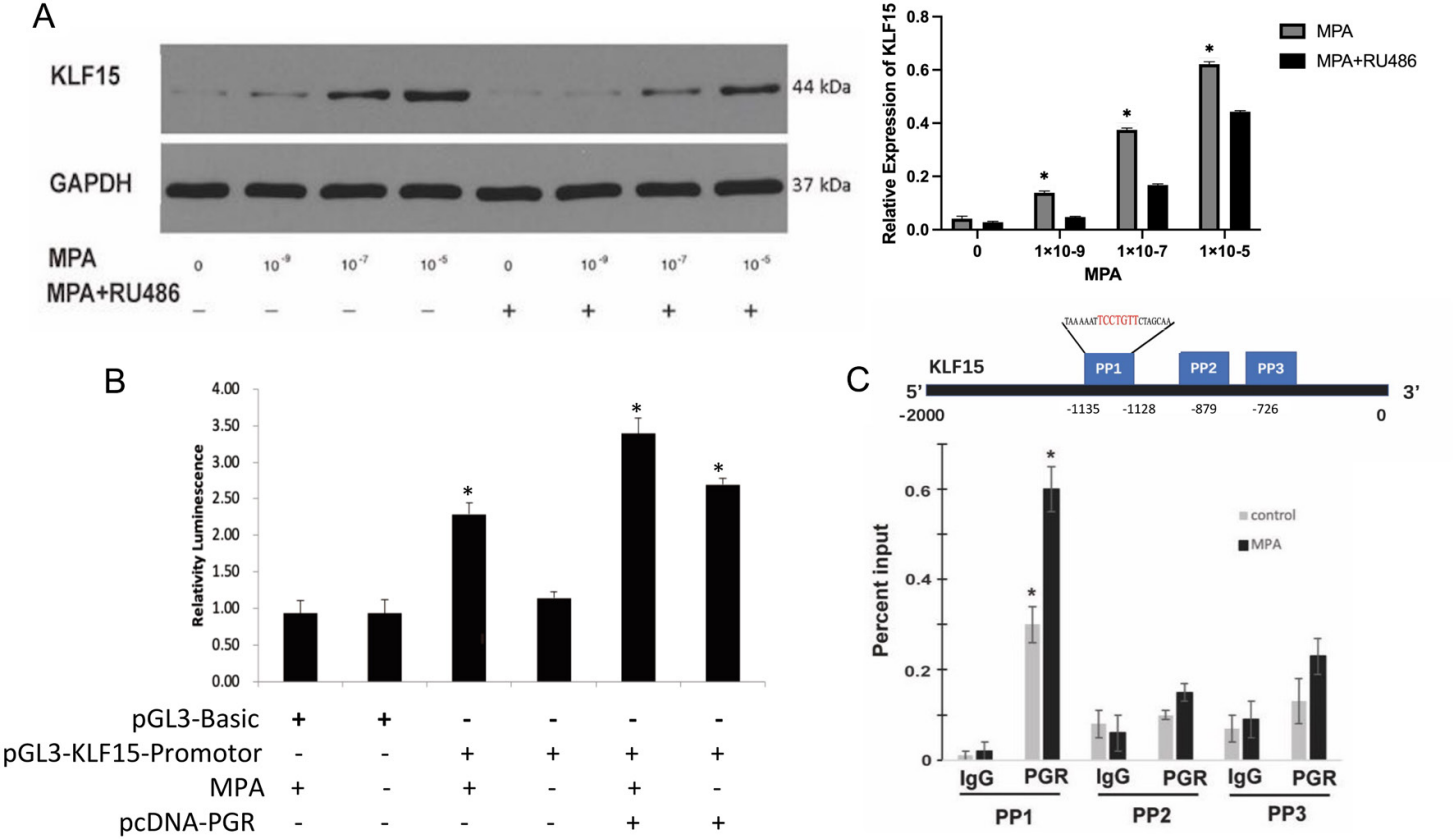
KLF15 is regulated by progesterone receptor. (A) The protein expression of KLF15 after treatment of different doses of MPA with or without RU486 (*n* = 4). (B) Luciferase assays of the activation potential of the region containing pGL3-KLF15 promoter in the presence of PGR overexpression plasmid, MPA, or PGR overexpression plasmid combined with MPA (*n* = 4). *P < 0.05, using one-way ANOVA. (C) Putative binding area of PGR on KLF15 promotor region and ChIP-qPCR analysis of recruitment of PGR to the putative binding area on KLF15 promoter region after MPA treatment (*n* = 3). *P < 0.05, using *t*-test. A full colour version of this figure is available at https://doi.org/10.1530/JOE-23-0319.

Progesterone is the key hormone in the establishment of endometrial receptivity. It has been demonstrated that progesterone resistance is the main cause of embryo implantation failure in EM patients with infertility (Burney *et al.* 2007). Considering that progesterone receptor (PGR) plays a crucial role in regulating endometrial receptivity, we examined the impact of progesterone and PGR on KLF15 expression. We treated Ishikawa cells with different doses of MPA with or without the progesterone receptor antagonist mifepristone (RU486). We observed a significant increase in KLF15 expression with the addition of MPA. However, stimulation of Ishikawa cells with RU486 (10 µM) significantly inhibited MPA-induced overexpression of KLF15 (Fig. 3A), indicating that KLF15 is mainly regulated by the progesterone–PGR signaling during the endometrial secretory phase.

**Figure 4:**
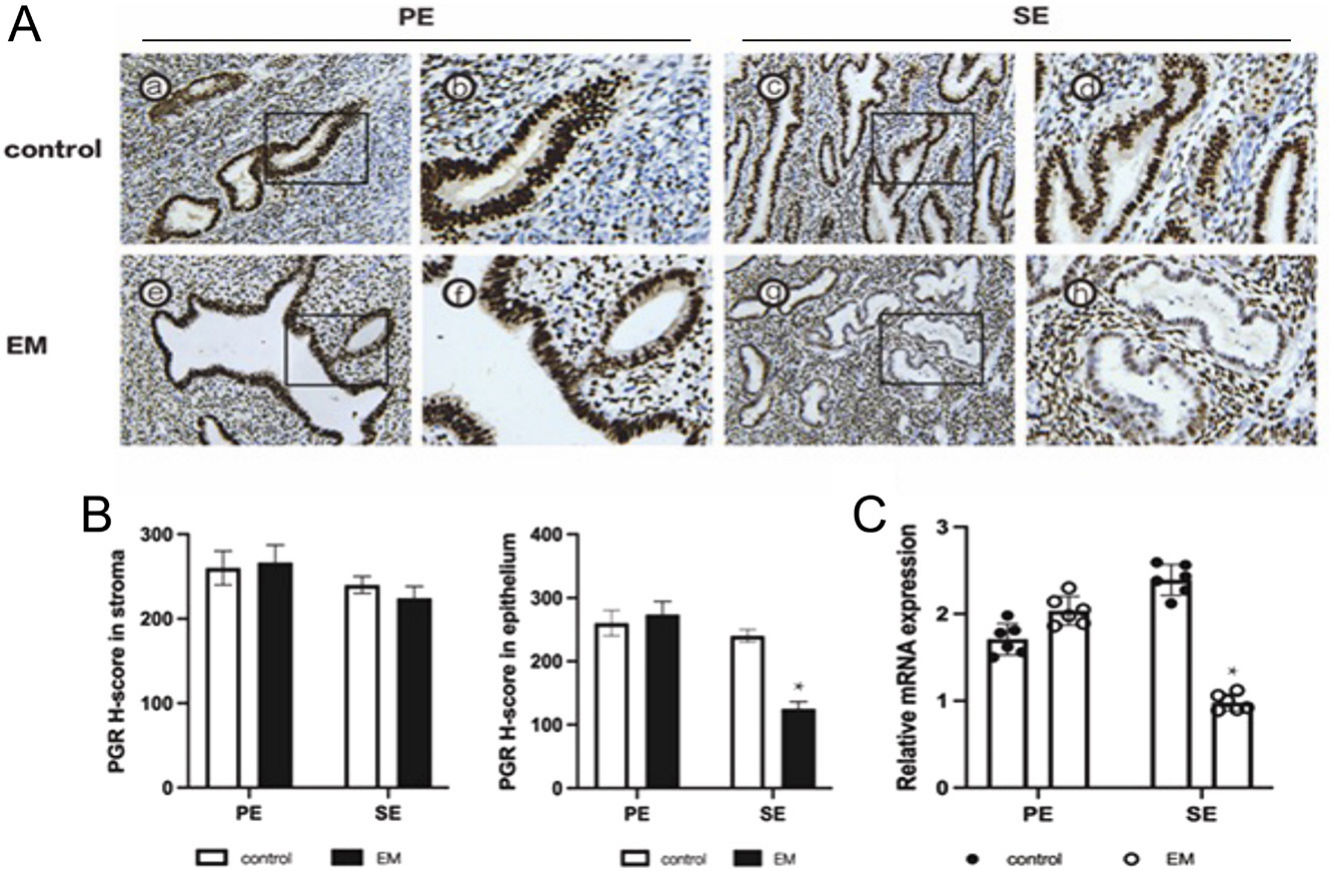
PGR expression in endometriosis. (A) Representative photomicrographs of PGR protein expression in EM and control group. The left photos were shown in 200× magnification, the right photos were shown in 400× magnification. (B) Immunohistochemical *H*-score of PGR proteins in proliferative (*n* = 6) and mid-secretory phase (*n* = 6) endometrium from women with (*n* = 12) or without (*n* = 12) endometriosis. (C) RT-qPCR analysis of PGR expression in different phases of endometrium in patients with (*n* = 12) or without endometriosis (*n* = 12). *P < 0.05. A full colour version of this figure is available at https://doi.org/10.1530/JOE-23-0319.

Considering that RU486 is also an antagonist of the glucocorticoid receptor, we conducted a luciferase activity assay and chromatin immunoprecipitation followed by qPCR (ChIP-qPCR) to further confirm the regulation between PGR and KLF15. The KLF15 promoter-pGL3 plasmid was transfected into Ishikawa cells, and the dual luciferase assay showed a significant increase in luciferase activity after MPA treatment, PGR overexpression, and MPA+PGR overexpression (Fig. 3B). We predicted potential binding sites between KLF15 and PGR using the PROMO website and designed primers for ChIP-qPCR at corresponding areas (Fig. 3C). ChIP-qPCR analysis showed prominent binding of PGR at the KLF15-PREs-BE locus (bp −1135 to −1128) using a PGR antibody after MPA treatment (Fig. 3C). Additionally, the *H*-score and mRNA expression of PGR in epithelial cells of eutopic endometrium of women with endometriosis were decreased compared to patients without endometriosis (Fig. 4). These findings suggest that the transcription of KLF15 may be directly regulated by PGR binding to the KLF15-PRE-BE sequence.

### KLF15 regulates endometrial EMT

**Figure 5:**
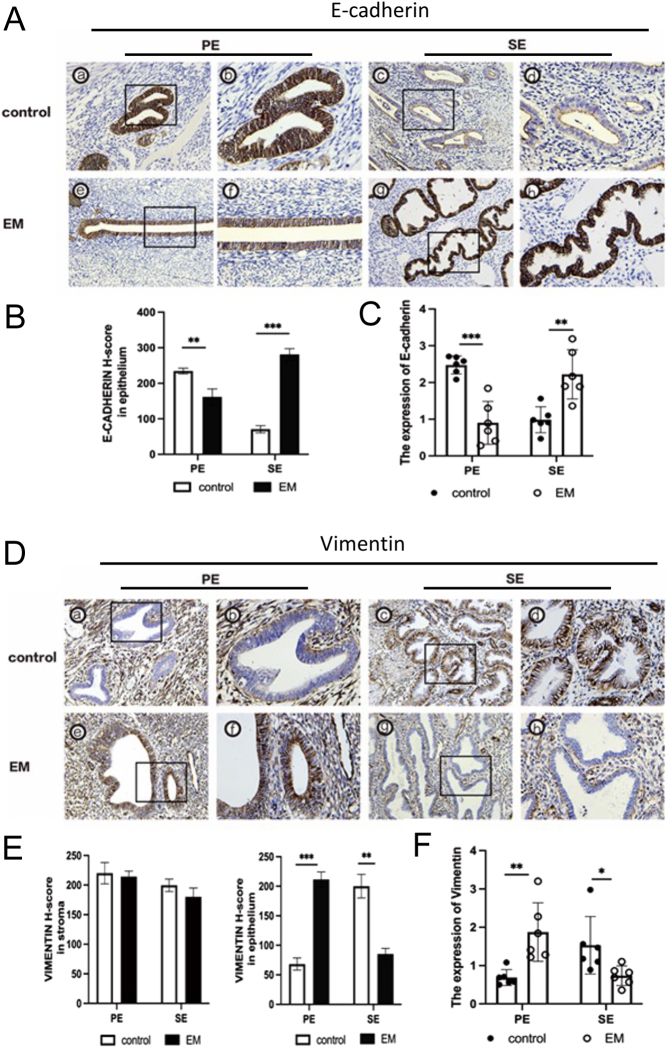
E-cadherin and vimentin expression in endometriosis. (A, D) Representative photomicrographs of E-cadherin and vimentin protein expression in EM and control group. The left photos were shown in 200× magnification, the right photos were shown in 400× magnification. (B, E) Immunohistochemical *H*-score of E-cadherin and vimentin proteins in proliferative and mid-secretory phase endometrium from women with (*n* = 12) or without endometriosis (*n* = 12). (C, F) RT-qPCR analysis of E-cadherin and vimentin expression in different phases of endometrium in patients with (*n* = 12) or without endometriosis (*n* = 12). *P < 0.05. **P < 0.01, ***P < 0.001. A full colour version of this figure is available at https://doi.org/10.1530/JOE-23-0319.

During embryo implantation, the maternal luminal epithelium undergoes EMT to accommodate the invading trophoblast. We investigated the expression of EMT markers (E-cadherin and vimentin) in women with endometriosis. In the control group, E-cadherin expression decreased and vimentin increased during the mid-secretory phase compared to the proliferative phase. However, in women with endometriosis, E-cadherin expression was upregulated, and vimentin was decreased in the mid-secretory phase compared to women without endometriosis (Fig. 5). These results suggest that EMT is impaired in the mid-secretory endometrium of women with endometriosis.

**Figure 6:**
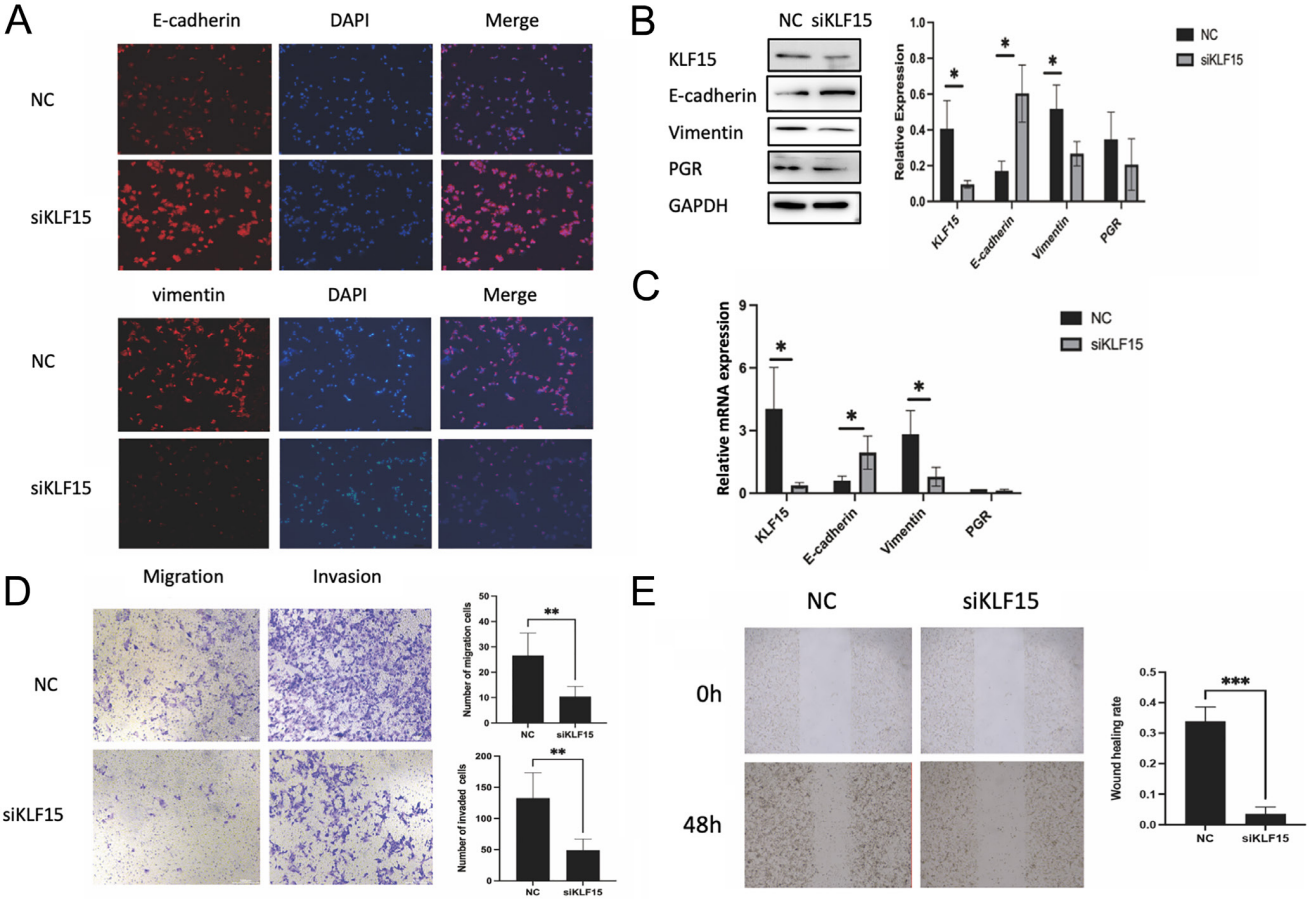
KLF15 regulates EMT of Ishikawa cells. (A) Representative images of E-cadherin and vimentin expression in Ishikawa cells transfected with KLF15 siRNA. (B, C) Western blot and RT-qPCR analysis of E-cadherin and vimentin expression after KLF15 knockdown (*n* = 4). (D) Chamber transwell assays of cellular invasion or migration (*n* = 6). (E) Wound healing assay analysis Ishikawa cells migration rate after KLF15 siRNA transfection (*n* = 6). *P < 0.05, **P < 0.01, ***P < 0.001, using *t*-test. A full colour version of this figure is available at https://doi.org/10.1530/JOE-23-0319.

Next, we examined the changes in EMT markers after downregulating KLF15 *in vitro*. Immunofluorescence staining showed that after KLF15 knockdown, E-cadherin staining significantly increased, while vimentin staining decreased in Ishikawa cells (Fig. 6A). The mRNA and protein expression of E-cadherin increased, and vimentin decreased after KLF15 knockdown (Fig. 6B and C). As EMT is closely related to cell invasiveness and migratory properties, scratch wound healing and transwell invasion assays were performed. We observed that fewer Ishikawa cells passed through the lower chamber, and the migratory and invasive potential was decreased after KLF15 knockdown (Fig. 6D and E). These results indicate that loss of KLF15 may induce a blockade in EMT, which is crucial for endometrial epithelial receptivity establishment.

### KLF15 promotes EMT via TWIST2

**Figure 7:**
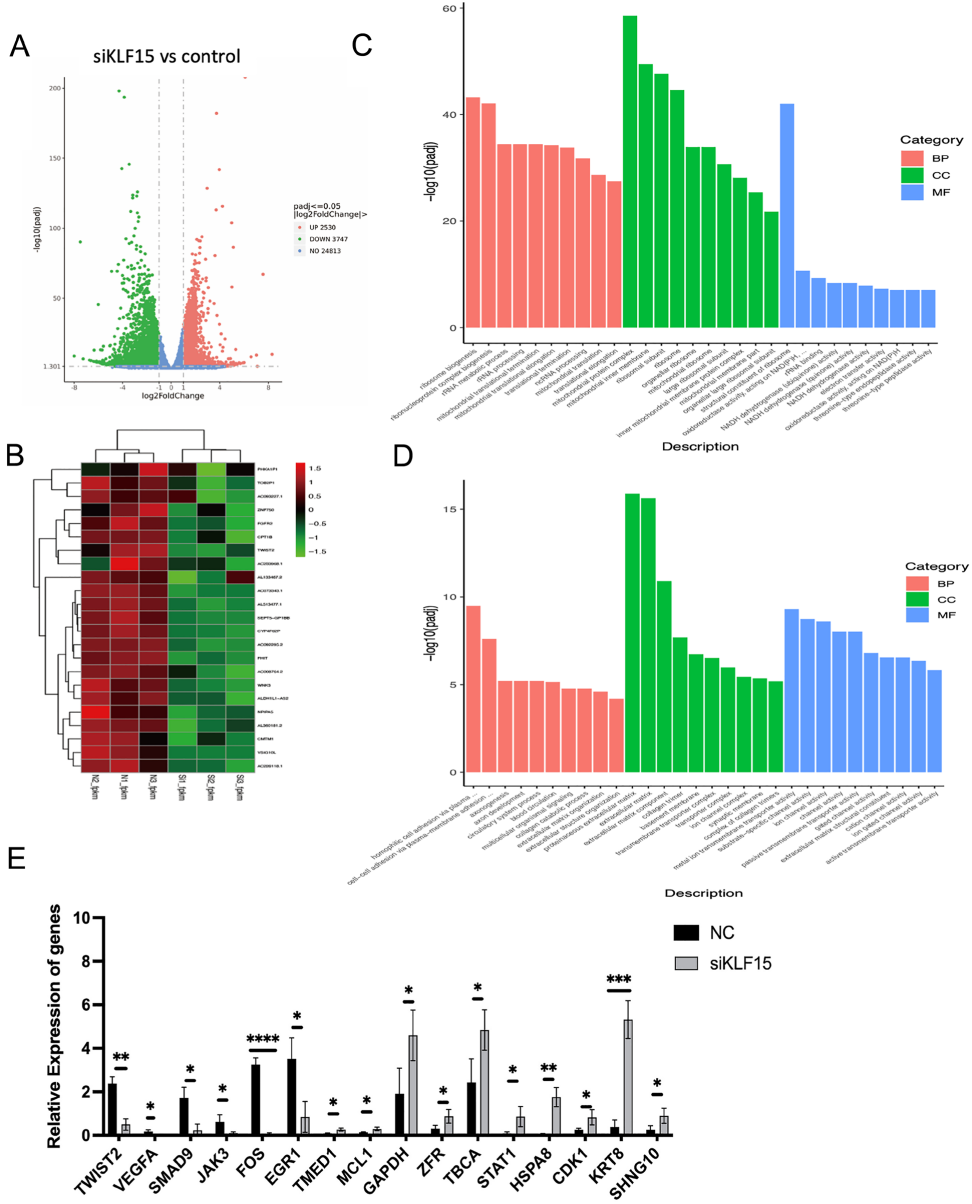
RNA-seq analysis of genes related to KLF15 downregulation. (A) Volcano diagram of upregulated and downregulated genes after KLF15 knockdown. (B) Heatmap of differential gene expression after KLF15 knockdown. Gene Ontology analysis revealed upregulated (C) and downregulated (D) gene function after KLF15 knockdown (*n* = 3). (E) RT-qPCR analysis of 16 DE gene expression (*n* = 3), *P < 0.05, *P < 0.01, **P < 0.001, ****P < 0.0001, using *t*-test. A full colour version of this figure is available at https://doi.org/10.1530/JOE-23-0319.

To identify the target gene of KLF15, we performed RNA-seq using Ishikawa cells transfected with or without KLF15-siRNA. After KLF15 knockdown, 2530 genes were upregulated, and 3747 genes were downregulated (Fig. 7A and B). Gene ontology analysis revealed that upregulated genes were related to ribonucleoprotein complex biogenesis and rRNA metabolic process (Fig. 7C), while downregulated genes were related to cell adhesion via plasma membrane molecules (Fig. 7D). We selected 16 genes for PCR validation, and the results were consistent with the RNA-seq. Among the 16 genes validated through RT-qPCR, TWIST2, VEGFA, and other genes have been identified in previous studies as being associated with endometrial receptivity (Cao *et al.* 2022). We focus on TWIST2, a positive regulator of EMT, which exhibited a significant decrease in expression (Fig. 7E).

**Figure 8:**
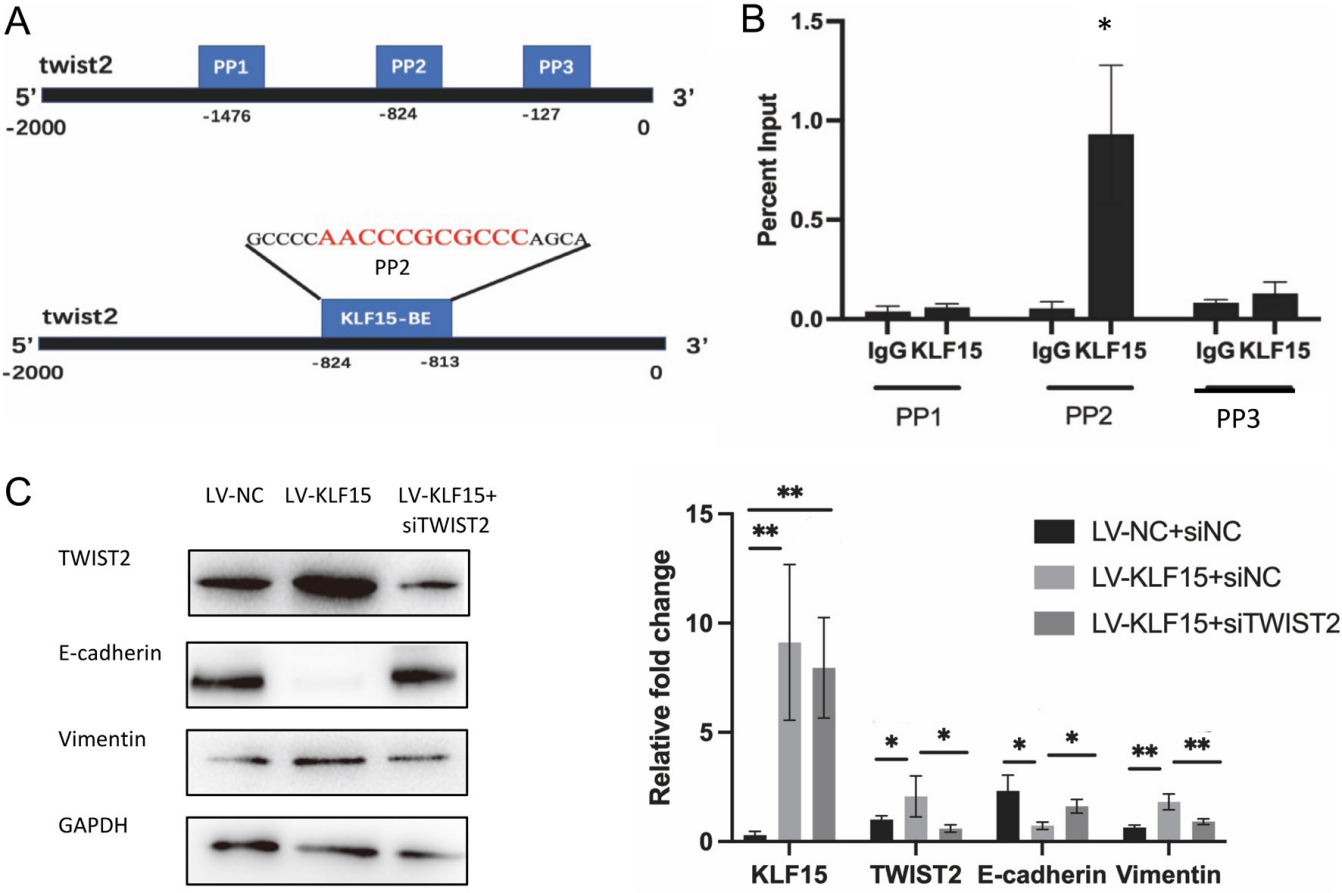
KLF15 modulates EMT via TWIST2 expression. (A) Putative binding area of KLF15 on TWIST2 promoter region. (B) Chromatin immunoprecipitation (ChIP) qPCR analysis of recruitment of KLF15 to PP2 of TWIST2 was increased (*n* = 3). (C) Knockdown of TWIST2 significantly inhibits KLF15 overexpression-induced EMT markers expression (*n* = 3). LV-KLF15: KLF15 overexpression. LV-NC, Negative control overexpression plasmid. *P < 0.05, **P < 0.01. A full colour version of this figure is available at https://doi.org/10.1530/JOE-23-0319.

To investigate whether KLF15 is a transcription factor of TWIST2, we predicted three possible binding sites of KLF15 in the TWIST2 promoter region (Fig. 8A). ChIP-qPCR assay showed that one specific binding site (PP2) on the TWIST2 promoter was critical for the recruitment of KLF15 (Fig. 8B). Overexpression of KLF15 led to upregulation of TWIST2, decreased E-cadherin, and increased vimentin expression (Fig. 8C and Supplementary Fig. 5A). Cell invasion and migration abilities were also significantly increased after KLF15 overexpression (Supplementary Fig. 6A). However, EMT changed inversely when TWIST2 was knocked down, as well as cell invasion and migration abilities (Fig. 8C, Supplementary Figs. 5B and 6B), suggesting that KLF15 promotes EMT by positively regulating TWIST2 expression. Furthermore, KLF15 overexpression promoted the attachment of JAR spheroids (mimicking blastocysts) to Ishikawa cells, indicating that KLF15 expression enhances embryo adhesion (Supplementary Fig. 7).

## Discussion

Poor endometrial receptivity is responsible for about two-thirds of implantation failure, whereas the quality of the embryo is responsible for only one-third of such cases (Lessey & Young 2019). The expression profile of endometrial genes and proteins in secretory endometrium was changed in EM patients (Xin *et al.* 2017, Anupa *et al.* 2019). To accept embryo implantation, epithelium barrier destruction, and cell structure remodeling were prerequisites (Wang & Dey 2006, Fukui *et al.* 2019). *In vitro* models for embryo implantation showed that 17-estradiol combined with progesterone induces EMT and promotes the attachment rate of JAR spheroids to Ishikawa cells (Uchida *et al.* 2012). During the secretory phase of the endometrium, there is a downregulation of the epithelial marker E-cadherin and an upregulation of the mesenchymal markers vimentin and N-cadherin (Gou *et al.* 2019, Oghbaei *et al.* 2022). In addition, adhesion molecules redistribute from the epithelial cell surface to plasma and lose their polarity. These changes may promote adhesion between the endometrium and the embryo (Whitby *et al.* 2020), and facilitate the implantation of the embryo into the endometrial stromal cell layer (Ran *et al.* 2020). In our study, we found that E-cadherin was increased and vimentin was reduced in the endometrium of patients with endometriosis, indicating that EMT was blocked during mid-secretory transformation in endometriosis.

The KLF family plays a pivotal role in orchestrating endometrial receptivity during embryo implantation. Studies reveal that specific KLF family members, including KLF12, exert a negative regulatory influence on human endometrial receptivity. Notably, KLF12 inhibits the decidualization of human endometrial stromal cells (hESC) by suppressing Nur77 expression (Huang *et al.* 2017). In eutopic endometrium from women with endometriosis, KLF9 mRNA was significantly reduced. Loss of KLF9 may underlie progesterone resistance in endometriosis (Pabona *et al.* 2012). However, most of the research about the KLF family was done in endometrial stromal cells, not epithelial cells. In our research, we found that there is a decrease in both KLF15 protein and mRNA expression in the eutopic endometrium of women with endometriosis, particularly during the mid-secretory phase. This may indicate a potential role of KLF15 in the development of endometrial receptivity and could contribute to the embryo implantation failure seen in women with endometriosis-associated infertility. Further experiments revealed that knockdown of KLF15 reversed the expression of EMT markers in Ishikawa cells. Through RNA-seq and ChIP-qPCR assays, we discovered that KLF15 directly binds to TWIST2 promoter regions and may act as a transcription factor for TWIST2, promoting EMT. Loss of KLF15 may reverse TWIST2-induced EMT in endometrial epithelial cells.

During EMT, there may be other changes in the main components of cell–cell connections, such as claudin and occludin, which were decreased, and cytoskeleton remodeling was similar to that seen in tumor cells during invasion and metastasis (Kardash *et al.* 2010, Choi *et al.* 2015, Whitby *et al.* 2020). The concentration of globular actin monomer, namely, G-actin, increases on the cell surface and aggregates into filamentous actin, which is called F-actin. This redistribution of actin disrupts the concentration of cortical actin and its regulatory proteins, transferring them to the edges of cells to form invasive pseudopodia in the front. All the changes would endow the cells with the capacity to migrate and invade (Kardash *et al.* 2010). Our results revealed that knockdown of KLF15 significantly reduced EMT, embryo implantation rate, as well as invasion and migration ability of endometrial epithelial cells. The reduced invasion and migration ability in the downregulated KLF15 cells reflected the blocked cellular remodeling, which could contribute to embryo implantation failure. Conversely, overexpression of KLF15 promoted invasion and adhesion ability. Embryo implantation involves the adhesion and invasion processes of trophoblast and luminal epithelial (LE) cells. During the proliferative phase, polar LE cells undergo plasma membrane remodeling, enabling interactions with trophoblast cells. Successful pregnancy requires embryo penetration through LE at the implantation site. Notably, LE tight junctions may influence the pattern of embryo penetration. The reduction in tight junctions from the onset of the luteal phase to the mid-luteal phase (or early pregnancy), potentially facilitates trophoblast passage (Whitby *et al.* 2020). In summary, EMT marker expression in glandular epithelial cells signifies structural remodeling during embryo implantation, involving changes in molecule expression and localization crucial for endometrial receptivity and embryo implantation.

Several studies have demonstrated progesterone resistance of eutopic endometrial cells in women with EM (Burney *et al.* 2007, Yilmaz & Bulun 2019, Reis *et al.* 2020). Progesterone is crucial to decrease inflammation in the endometrium, abnormal progesterone signaling results in a proinflammation phenotype. Conversely, chronic inflammation may induce a progesterone-resistant state (Patel *et al.* 2017, Chi *et al.* 2020). Our results indicated that progesterone receptor (PR) expression was not affected by knockdown of KLF15, but RU486 significantly reduced KLF15 expression, along with EMT markers. This suggests that KLF15 is regulated by PR and is involved in progesterone–PR signaling. The decreased expression of KLF15 suggests that loss of PR signaling may delay the attainment of receptivity.

**Figure 9:**
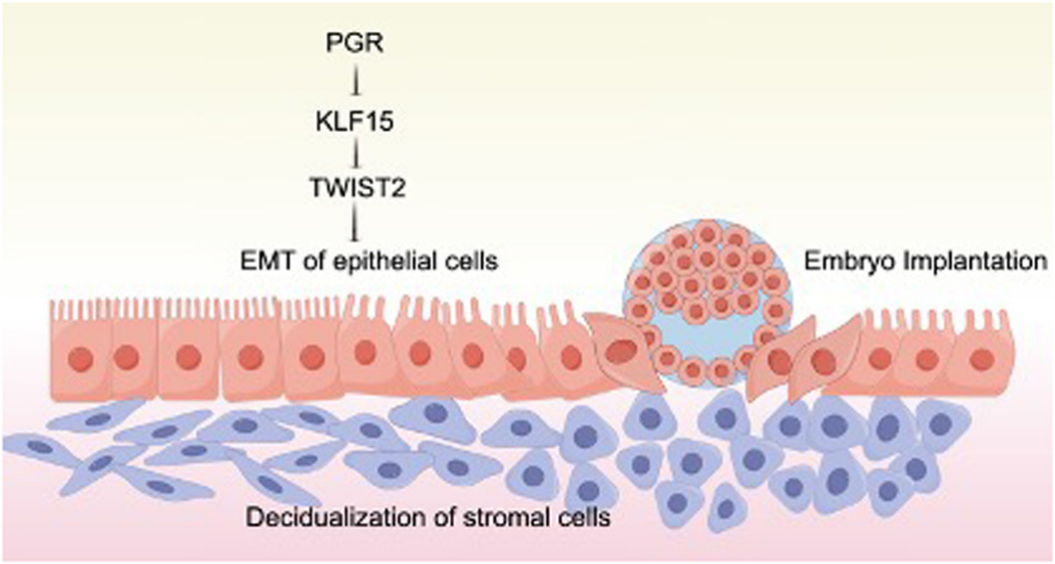
KLF15 is regulated by PGR, and KLF15 promotes TWIST2 induced EMT of endometrial epithelial cells during embryo implantation. A full colour version of this figure is available at https://doi.org/10.1530/JOE-23-0319.

Our research has revealed crucial insights into the involvement of KLF15 in endometriosis-related infertility. We observed the inhibition of EMT in the endometrial epithelial cells of the mid-secretory endometrium in women with endometriosis. Additionally, we identified PGR as a regulator of KLF15 expression, and KLF15 was found to directly bind to TWIST2 promoter regions, thereby promoting the expression of TWIST2-induced EMT markers (Fig. 9). The absence of KLF15 may reverse TWIST2-induced EMT in endometrial epithelial cells, potentially impairing endometrial receptivity and influencing the adhesion between embryos and endometrial epithelial cells. These findings have significant clinical implications as they enhance our understanding of why endometrial receptivity is compromised in women with endometriosis. In future investigations, it might be possible to explore new therapies for nonreceptive endometrium of EM patients.

## Supplementary Materials

Supplementary Figure 1: Establishment of endometriosis rat model. （A）The construction method of the endometriosis rat model. (B) Rat uterus. (C) Uterine tissue was sutured to the abdominal wall.

Supplementary Figure 2: Ectopic endometrium transplanted foci. (A) Ectopic transplanted uterus tissue in the mesentery. (B) Ectopic transplanted uterus tissue in the abdominal wall. The two sites formed cystic structures containing clear fluid. (C) Endometrial glands and stroma could be seen under the microscope of the transplanted lesions in the EM group. (D)No obvious lesions in the abdominal wall of the sham operation group, and no endometrial glands and interstitial structures were observed under the microscope (20x objective).

Supplementary Figure 3: KLF15 expression in EM rat model. (A) Representative photomicrographs of KLF15 protein expression in EM, sham, and control rat groups. Photos were shown in 400×magnification. (B) Immunohistochemical analysis of KLF15 intensity in eutopic endometrium on gestation days 1, 5, and 7 from endometriosis rats compared to control and sham group rats(n=6). (C)KLF15 mRNA expression in eutopic endometrium on gestation days 1,5 and 7 from EM rat compared to control and sham group rats(n=6). *P < 0.05, using one-way ANOVA.

Supplementary Figure 4: (A) Representative photomicrographs of JAR spheroids attachment to Ishikawa cells with KLF15 knockdown. (B) Percentage of JAR spheroids attachment reduced after KLF15 knockdown(n=4). **P < 0.01, using t-test.

Supplementary Figure 5: (A) Representative E-cadherin and Vimentin expression images in Ishikawa cells transfected with KLF15-overexpression plasmid. (B) Representative E-cadherin and Vimentin expression images in Ishikawa cells transfected with KLF15-overexpression plasmid with or without TWIST2 siRNA transfection.

Supplementary Figure 6: (A)Chamber transwell assays of cellular invasion or migration after KLF15 overexpression plasmid transfection(n=6). (B)Chamber transwell assays of cellular invasion or migration after KLF15 overexpression plasmid transfection with or without siTWIST2 transfection(n=6). (C) Wound healing assay analysis Ishikawa cells migration rate after KLF15 overexpression plasmid transfection(n=6). *P < 0.05, **P < 0.01, ***P<0.001. using t-test.

Supplementary Figure 7: (A) The representative photomicrographs of JAR spheroids attachment to monolayer Ishikawa cells after KLF15 overexpression. (B) The attachment number of JAR spheroids was elevated after KLF15 overexpression(n=4). *P < 0.05, using t-test.

## Declaration of interest

The authors declare that there is no conflict of interest that could be perceived as prejudicing the impartiality of the study reported.

## Funding

This work is supported by the National Nature Science Foundation of China (grant no. 81771543), the Health Commission of Hubei Province (grant no. WJ2021F050), and Fundamental Research Funds for Central University (grant no. 2042021kf0162).

## Availability of data and materials

The datasets analyzed during the current study are available from the corresponding author on reasonable request.

## Ethical conduct of research

The study was approved by the Ethics Committee of Dongfeng General Hospital (LW-2022-021). Written informed consent was obtained from patients. All animal experiments were approved by the Institutional Animal Care and Use Committee and following the 3R principle of experimental animals.

## Author contributions

YH and ZW designed the study and performed experiments. YX was a major contributor to writing and revising the manuscript. BL and LK recruited patients and performed the examination of the endometrium. YZ conceived the study and revised the draft of the manuscript.
