# Supplementary figures and images for: Loss of KLF15 impairs endometrial receptivity by inhibiting EMT in endometriosis
Source: J Endocrinol. 2024 Apr 17;261(2):e230319. doi: 10.1530/JOE-23-0319 (PMC11056958; doi:10.1530/JOE-23-0319)

Laparotomy  
Resection of uterus

Autotransplantation  
onto peritoneum

(A)

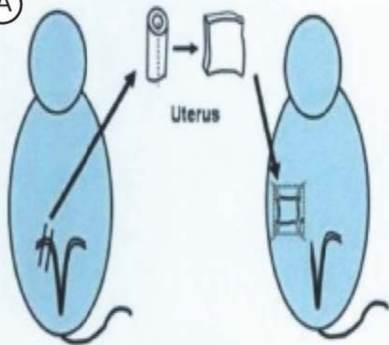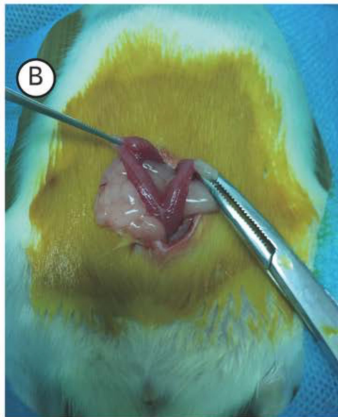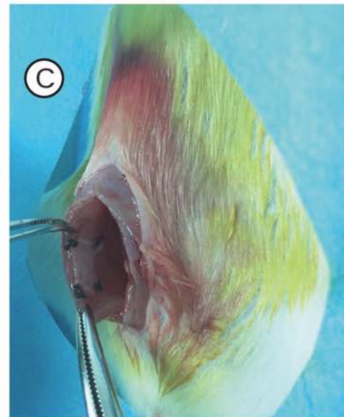

Supplement: Supplementary Figure 1: Establishment of endometriosis rat model. （A）The construction method of the endometriosis rat model. (B) Rat uterus. (C) Uterine tissue was sutured to the abdominal wall. [file supplementary_figure_1.pdf]

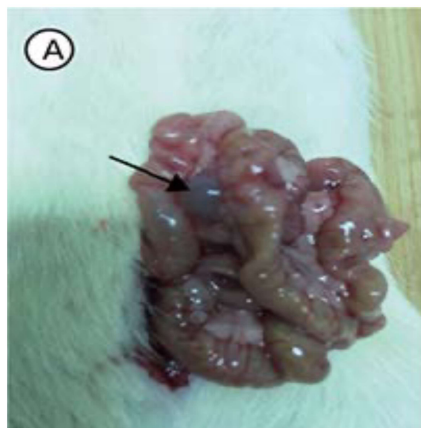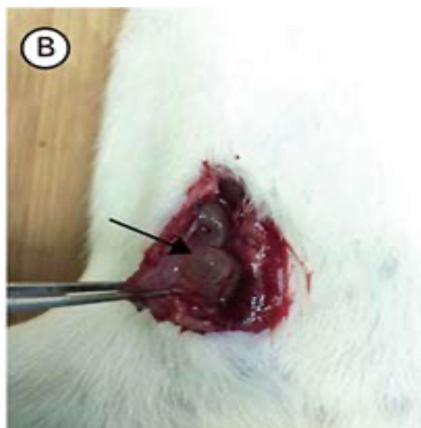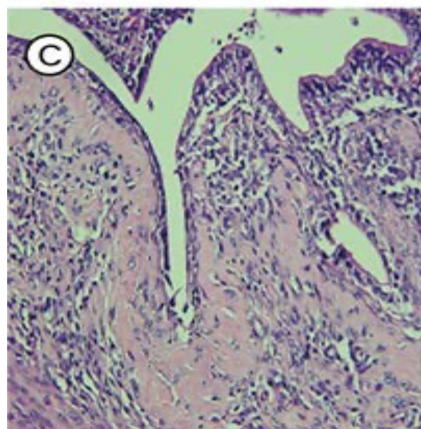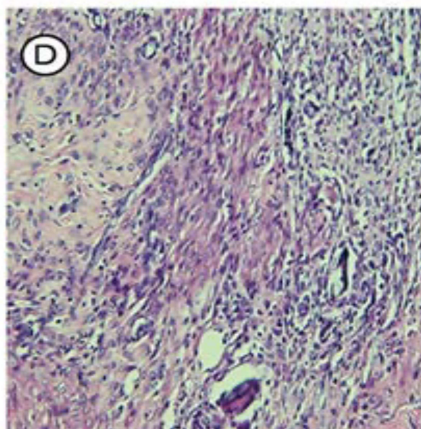

Supplement: Supplementary Figure 2: Ectopic endometrium transplanted foci. (A) Ectopic transplanted uterus tissue in the mesentery. (B) Ectopic transplanted uterus tissue in the abdominal wall. The two sites formed cystic structures containing clear fluid. (C) Endometrial glands and stroma could be seen under t [file supplementary_figure_2.pdf]

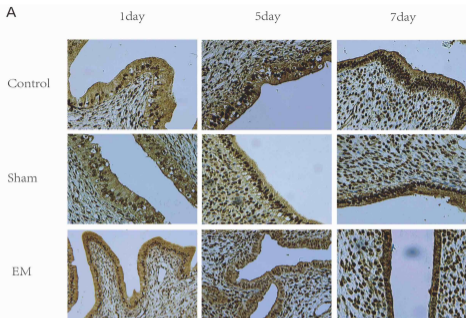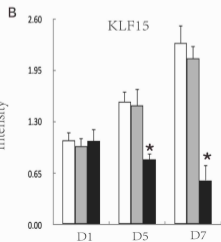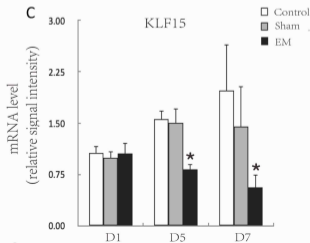

Supplement: Supplementary Figure 3: KLF15 expression in EM rat model. (A) Representative photomicrographs of KLF15 protein expression in EM, sham, and control rat groups. Photos were shown in 400×magnification. (B) Immunohistochemical analysis of KLF15 intensity in eutopic endometrium on gestation days 1, 5, an [file supplementary_figure_3.pdf]

**A****JAR cells Spheroids****Ishikawa cells****Merge****NC**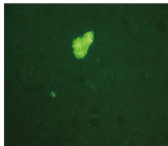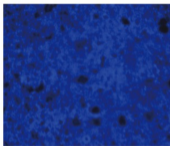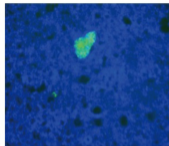**siKLF15**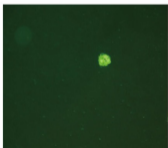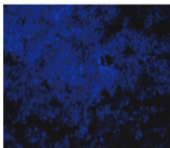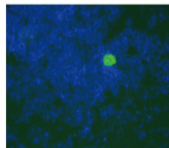**B**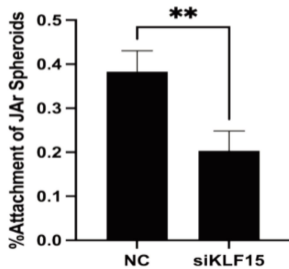

Supplement: Supplementary Figure 4: (A) Representative photomicrographs of JAR spheroids attachment to Ishikawa cells with KLF15 knockdown. (B) Percentage of JAR spheroids attachment reduced after KLF15 knockdown(n=4). **P < 0.01, using t-test. [file supplementary_figure_4.pdf]

**A**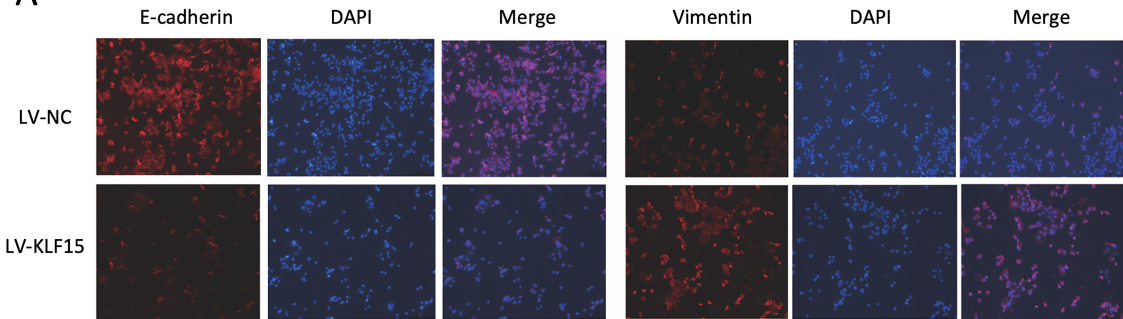

LV-KLF15

**B**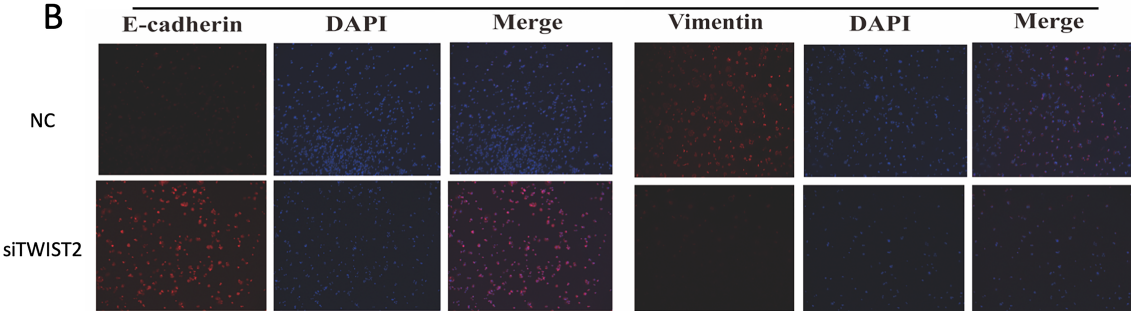

Supplement: Supplementary Figure 5: (A) Representative E-cadherin and Vimentin expression images in Ishikawa cells transfected with KLF15-overexpression plasmid. (B) Representative E-cadherin and Vimentin expression images in Ishikawa cells transfected with KLF15-overexpression plasmid with or without TWIST2 si [file supplementary_figure_5.pdf]

**A**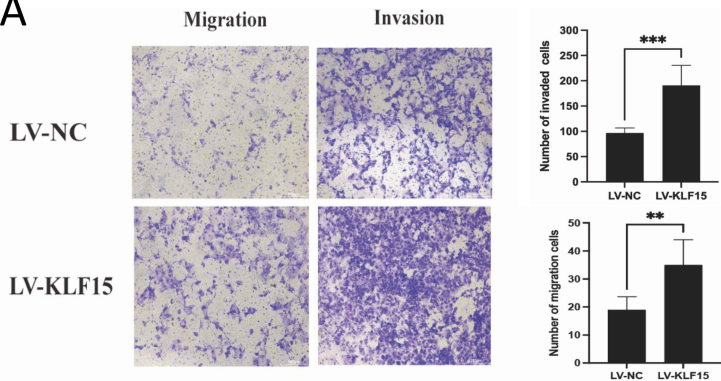**B**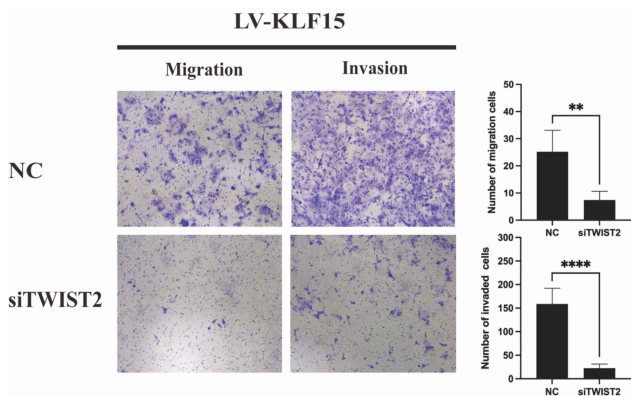**C**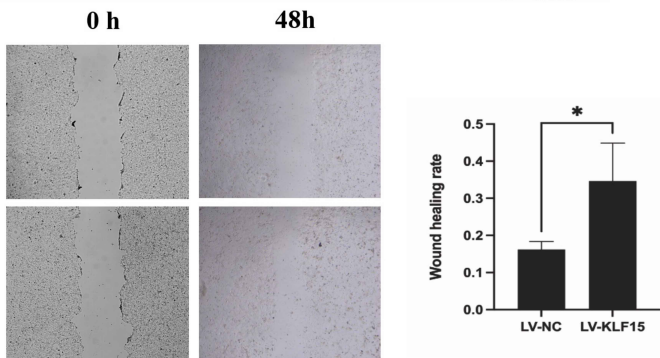

Supplement: Supplementary Figure 6: (A)Chamber transwell assays of cellular invasion or migration after KLF15 overexpression plasmid transfection(n=6). (B)Chamber transwell assays of cellular invasion or migration after KLF15 overexpression plasmid transfection with or without siTWIST2 transfection(n=6). (C) Wo [file supplementary_figure_6.pdf]

**A**

JAR cells Spheroids

Ishikawa cells

Merge

LV-NC

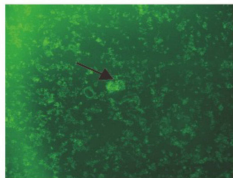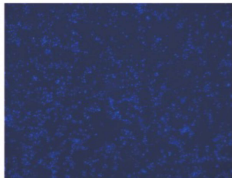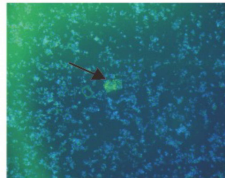

LV-KLF15

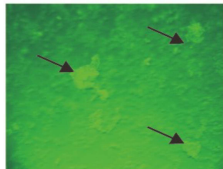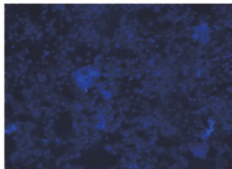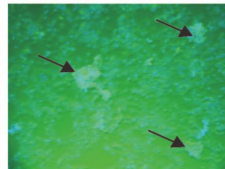**B**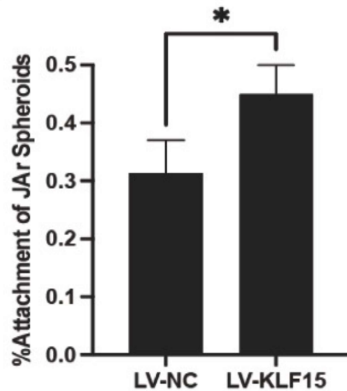

Supplement: Supplementary Figure 7: (A) The representative photomicrographs of JAR spheroids attachment to monolayer Ishikawa cells after KLF15 overexpression. (B) The attachment number of JAR spheroids was elevated after KLF15 overexpression(n=4). *P < 0.05, using t-test. [file supplementary_figure_7.pdf]
